# Teaching and Learning Process of Decision-Making Units in Talented Young Players From U-10 to U-14

**DOI:** 10.3389/fpsyg.2020.00600

**Published:** 2020-04-22

**Authors:** Juan Carlos Pastor-Vicedo, Alejandro Prieto-Ayuso, Onofre Ricardo Contreras-Jordán, Filipe Manuel Clemente, Pantelis Theo Nikolaidis, Thomas Johannes Rosemann, Beat Knechtle

**Affiliations:** ^1^Department of Didactics of Musical, Plastic and Physical Education, Faculty of Education, University of Castilla-La Mancha, Albacete, Spain; ^2^Department of Didactics of Musical, Plastic and Physical Education, Faculty of Education, University of Castilla-La Mancha, Cuenca, Spain; ^3^Albacete Balompiè S.A.D., Albacete, Spain; ^4^Escola Superior Desporto e Lazer, Instituto Politécnico de Viana do Castelo, Rua Escola Industrial e Comercial de Nun’Álvares, Viana do Castelo, Portugal; ^5^Instituto de Telecomunicações, Delegação da Covilhã, Covilhã, Portugal; ^6^Exercise Physiology Laboratory, Nikaia, Greece; ^7^Institute of Primary Care, University of Zurich, Zurich, Switzerland; ^8^Medbase St. Gallen Am Vadianplatz, St. Gallen, Switzerland

**Keywords:** gifted, exercise performance, sport, physical education, curriculum, constraint-led approach, tactical

## Abstract

There are multiple factors that have been studied for talent identification (TI) with regard to sport performance, such as physical and physiological parameters; psychological, social, and contextual parameters; and technical–tactical parameters. However, despite the importance of these indicators for reaching the elite, new trends seem to assure that one of the key elements in a young player is decision making (DM). Thus, in the last decades, research DM in young players has increased. Nevertheless, very little has been done in relation with DM and talented players. The purpose of this study was to analyze and compare the effectiveness, number, and duration of DM units (DMUs) of three groups of talented young players in U-10, U-12, and U-14 levels. Ninety-seven youth players participated in the study. A total of 1,087 actions were analyzed. The Nomination Scale for Identifying Football Talent was utilized to screen the talent pool (*N* = 18), and the Game Performance Evaluation Tool was used for analyzing the 1,087 actions completed. The results showed that the effectiveness has to be more than 80% for children to be considered talented. Moreover, a greater effectiveness of DMUs was shown in older age groups. The game speed also increased with age. It was revealed that U-12 did not follow the progression in the decisional demands in the formative stages. It is highlighted, therefore, the necessity of reviewing the organizational aspects in the U-12 age group, related mainly to the size of the pitch and the number of players, because it does not follow the same progression in regard to decisional demands. Future studies should follow this study with the U-16, U-18, and U-23 age groups, with the purpose of knowing the effectiveness, duration, and number of DMUs in older age groups. Furthermore, policy makers and teachers/coaches from both educational and soccer context must take these results into account, with the purpose of adjusting the teaching and learning process of talented children in sport.

## Introduction

There are multiple factors that have been studied for talent identification (TI) in soccer with regard to sport performance, such as physical and physiological parameters (Zibung et al., 2016; Murr et al., 2018) psychological, social, and contextual parameters (Höner and Feichtinger, 2016) and technical–tactical parameters (Woods et al., 2016; Sgrò et al., 2018). However, despite the importance of these indicators for reaching the elite (Dellal et al., 2011; Liu et al., 2015), new trends seem to assure that one of the key elements in a young soccer player is decision making (DM) (Serra-Olivares et al., 2016). An effective DM training from childhood possibly helps young players to solve problems that require a quick response in a short period of time (Collins and MacNamara, 2012). That process would allow young players to be successful in those decisional demands call for a specific sport, reaching the top level from school age (MacNamara and Collins, 2017). One of the theories that want to develop the DM in sports is called *constraints-led approach* (CLA). This theory is based on a learner–environment-centered approach in which participants are encouraged to modify constraints to develop perception–action couplings, focusing on the nature of learning activities (Renshaw et al., 2016). Regarding the teaching and learning process of DM, Mitchell et al. (2003, 2013) stated that suitable progression is based on the type of game, from target games, followed by striking and fielding games, and net/wall games, to invasion games (IGs). Thus, owing to its tactical complexity (format of the game and number of players), IGs are the most suitable for improving DM in children (Memmert and Harvey, 2010), especially on the game knowledge and game playing, compared with a traditional teaching style (Gray and Sproule, 2011). The level of complexity can be determined by the maximum number of players (Gutiérrez et al., 2014) and the format of the game (Serra-Olivares et al., 2016). These constraints (size of pitch and number of players) will change the objective of the task, adding, or eliminating complexity (Renshaw et al., 2019). In sports, for example, Vilar et al. (2012) investigated how the locations of the goal and ball constrain the pattern-forming dynamics of attacker–defender dyadic systems. The results help us to understand interpersonal interactions in team sports by explaining how attackers and defenders use information about their relative positioning to the goal and the ball to perform successfully. In soccer, smaller formats (2 vs. 2 or 3 vs. 3) reduce the variability of the game and increase the individual participation of each player (González-Víllora et al., 2011). Bigger formats increase tactical complexity by increasing the variability of actions and the possibilities of play (Clemente et al., 2014a, b). The tactical goals of these kind of games are as follows: (a) scoring on offense: keeping possession, penetrating and attacking, and transitioning from offense to defense; (b) preventing scoring on defense: defending space, defending the goal, and winning the ball; and (c) starting and restarting play: beginning the game, restarting from the sideline and/or endline, and restarting from violations (Mitchell et al., 2013).

In the last decades, the importance of researching DM has been increased. One example of that is the number of instruments built for measuring DM in young players (González-Víllora et al., 2015b). For example, Game Performance Assessment Instrument (GPAI) (Oslin et al., 1998), the Performance Assessment in Team Sports (TSAP) (Gréhaigne et al., 1997), the Procedural Tactical Knowledge Test (KORA) proposed by Roth and Kroeber (2002) and validated by Memmert (2002), the Game Performance Evaluation Tool (GPET) (García-López et al., 2013), and the System of Tactical Assessment in Soccer (FUT–SAT) (Costa et al., 2011).

GPET has been widely used in young players. For example, Sánchez-Mora et al. (2011) aimed to gauge the game performance in a modified IG in U-10, concluding that tactical knowledge and performance by means of a modified IG is important in implementing quality games-teaching program. Gutiérrez et al. (2014) investigated the game performance during an IG in U-14. Also, the technical–tactical knowledge of young soccer players in age groups U-10 (González-Víllora et al., 2010, 2015a), U-12 (González-Víllora et al., 2011, 2015a; Práxedes et al., 2018), and U-14 (González-Víllora et al., 2013, 2015a) has been investigated. Overall, the results of these studies highlighted a great importance of the cognitive aspects in game performance of young players, as well as the necessity to consider different tactical contexts for teaching and learning processes (Stolz and Pill, 2014; Penney and McMahon, 2016; Jarrett and Light, 2018).

In short, the training of DM seems to be essential in the development of young players (Bocarro et al., 2008; Giblin et al., 2014; Sarmento et al., 2018). Nevertheless, few researches have aimed to show the DM performance in talented young soccer players. Thus, with the purpose of describing the level of different age groups and adjusting the pedagogical strategies to optimize the teaching and learning process, the aim of this study was to compare the effectiveness, number, and duration of DM units (DMUs) of different age groups (U-10, U-12, and U-14) of talented young soccer players. We expect that there will be more DMUs in older age groups. Moreover, more experienced players will make quicker and right decisions, so the results obtained will be useful not only for soccer coaches but also for physical education (PE) teachers in order to adjust the teaching and learning process of DM in their pupils during PE lessons of sport initiation.

## Materials and Methods

### Study Design

The study design was inferential, correlational, and transversal (Montero and León, 2007). It is inferential because we aimed to extract conclusions applicable to other population. It is correlational because we pretended to establish relationship between DM/skills and age group. Finally, it is transversal because it has been done in a specific point of time.

Soccer players from a professional football club took part in the study. This football club runs in the second division of the Spanish League. An instrument [Nomination Scale for Identifying Football Talent (NSIFT)] was administered for selecting those talented players. Then, the game performance of these talented players was analyzed, establishing differences among age groups (U-10, U-12, and U-14) in regard to technical and tactical actions.

### Participants

We analyzed 1,087 actions of the offensive phase of 18 talented players from the U-10 to U-14 age group (see Table 1) in an IG: soccer.

**TABLE 1:** Distribution of the sample.

**Age group**	**Team**	**Number of players**	**Sessions of training**	**Hours of training**	**Age**	**Soccer playing years**	**Weight (kg)**	**Height (m)**	**Talent pool**
U-10	A	14	2/week	1/session	9.97 ± 0.18	4	33.37 ± 8.12	1.38 ± 0.07	6
	B	16							
U-12	A	13	2/week	1/session	11.46 ± 0.50	6	40.75 ± 7.50	1.50 ± 0.08	6
	B	16							
U-14	A	19	3/week	1/session	13.41 ± 0.55	8	51.36 ± 7.31	1.63 ± 0.08	6
	B	19							
Total	–	97	–	–	11.70 ± 1.51	–	42.38 ± 10.66	1.51 ± 1.20	18

**FIGURE 1:**
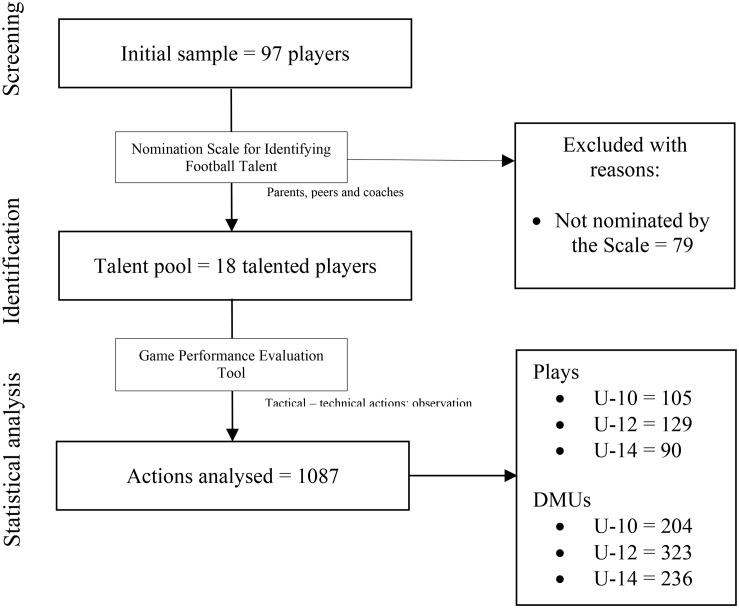
Flow diagram of participants’ selection. DMUs, decision-making units.

These talented players were selected from an initial sample formed by 97 young soccer players of the formative stages of a professional team that belongs to the second division of the Spanish League. Eighteen players were selected (see Figure 1) according to the 15% rule, which states that this percentage represents the talent pool of a sample (Gagné, 2015). The inclusion criterion was to have scored among the top 15% in the NSIFT instrument (more detailed in the *Instruments* section). The exclusion criterion was to have not scored among the top 15% in the instrument.

The club and the parents of the players gave written informed consent. The research project was fully approved by the Ethics Research Committee of the University of Castilla-La Mancha (Spain). The research has been developed under the recommendations of the Declaration of Helsinki.

### Instruments

In order to screen the talent pool, the NSIFT was used (Prieto-Ayuso et al., 2017). The NSIFT is a validated instrument that helps to select talented players through the evaluation of three dimensions: (1) tactical-technical skills, (2) creativity, and (3) commitment. The scale is a 13-item questionnaire:

1. Interprets the coach’s instructions correctly.
2. Usually anticipates play.
3. Generally makes the right decision.
4. Executes skills very quickly.
5. Able to read the game clearly and quickly.
6. Has good positional sense.
7. Knows where their teammates are on the pitch.
8. Makes an effort in matches and training.
9. Keen to learn and develop.
10. Able to concentrate in matches and/or training.
11. Possesses a winning mentality.
12. Has a positive attitude.
13. Willing to take on responsibilities.

There are no negative items in the scale. On the basis of the research conducted by Rogers (2002) in an educational context, parents fulfill the questionnaire through a Likert scale, from 1 (no correspondence) to 5 (very forthcoming). The highest punctuation is 65 points, and the lowest one is 13 points. Players nominated who is the best player in each point. The triangulation of the information derived from parents, experts, and players showed who the most talented players are. The internal consistency of NSIFT among stakeholders for this research scored 0.85 points (Cronbach alpha).

To assess DM, GPET observation instrument (García-López et al., 2013) was utilized. It is based on the DMU and plays. A DMU is the technical–tactical actions made by an attacking player in each play (e.g., pass the ball, run forward, and keep the ball). And the plays are the period since the team recovers the ball until they lose the ball.

**FIGURE 2:**
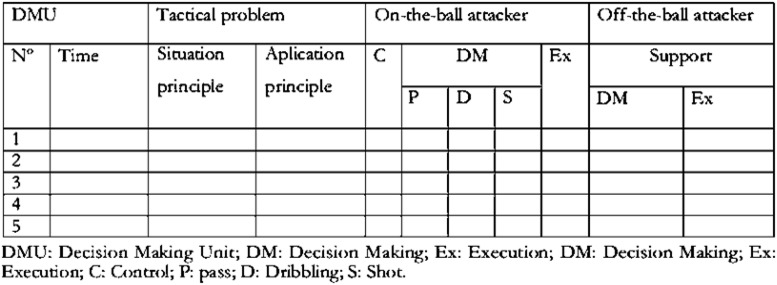
Game Performance Evaluation Tool (GPET) data sheet.

GPET evaluates game performance at two different levels. The first level evaluates how the players’ actions adapt to the tactical principles (Bayer, 1992): keeping possession of the ball (1A), advancing toward the opponent’s goal (2A), and scoring a goal (3A). At the second level, GPET separates the cognitive components from the DM and motor skills (control, passing, dribbling, shooting, offensive variable, and getting free). Figure 2 shows the GPET data sheet (García-López et al., 2013).

It a value of 1 was assigned to appropriate decisions and 0 to inappropriate decisions. The percentage of successful decisions was calculated individually for each participant. The total number of these decisions was divided by the sum of the number of the total of decisions and multiplied by 100. The criteria that were considered to assess if DM taken was successful or unsuccessful are specified in García-López et al. (2013).

The main researcher was trained in the use of GPET during eight periods of 1 h. After the training, intra-observer reliability was measured, with the 3 vs. 3 video record being analyzed twice. Two weeks elapsed between the first and second notations, in order to minimize bias between codings. Spearman’s rho rank correlation determined the researcher’s ability to reproduce results of this ordinal data. According to Losada and Arnau (2000), inter-observer reliability was measured by conducting ANOVA (kappa test), with the coding of both researchers (one author of GPET and the main researcher) being compared.

### Procedure

First, the approval of the Ethics Research Committee was received from the University. Also, the parents of the participants gave their informed consent about the participation of their sons in the study. Once the consent was collected, we conducted the TI process with all the potential participants (*N* = 97) in order to select the talent pool with the best 15% of players (Gagné, 2015). It was administered to the stakeholders of talent development: players, parents, and experts (coaches).

The main researcher was placed in the press center of the academy, owing to the adequate conditions of the room. The players completed the scale one by one with the main researcher, during a session training. It took 3 weeks to have all the questionnaires of players completed. They evaluated their teammates. The coaches completed the scale at the end of a session training. The main researcher gave the questionnaires to them, the coaches read and solved doubts, and then they completed the scale. Coaches evaluated their players, one by one. It took 1 week to have all the questionnaires of coaches completed. Finally, the main researcher met with the parents of each age group. They read the scale, solved the doubt, and then completed the questionnaires. Parents evaluated their sons. It took 3 weeks until all the questionnaires of parents were collected.

When the talent pool was formed, six full matches were recorded randomly of the official league in each age group (Table 2). One game was selected randomly per player. All the 18 players belong to the starting lineup. Owing to the schedule needs, these matches took place at the end of the season, and they were all recorded in 1 month, from the first until the last match observed. We used a Sony HDR-AS100VR Action Cam Full HD camera, DSK-Sony battery, Samsung memory card, and Cullmann tripod. The camera was located in the highest accessible point of the stadium in which each match took place, in order to follow the indications of GPET related to the necessity of recording the matches in the foreground. After recording the matches, they were downloaded in an HP 6730s computer. Then, the matches were analyzed during the first 10 min, according to the indications of GPET (García-López et al., 2013). The GPET sheet was used for analyzing the matches, and then this information was written in IBM SPSS Statistics, version 24.0. Once the data were included in SPSS, the analysis was conducted.

**TABLE 2:** Distribution of matches recorded.

**Age group**	**Number of matches recorded**	**Number of players**	**Field size (m)**	**Match duration (min)**	**Break time duration (min)**
U-10	2	8 vs. 8	45 × 30	50	10
U-12	2	8 vs. 8	45 × 30	60	10
U-14	2	11 vs. 11	90 × 60	70	10

### Data Analysis

First, means (*M*) and standard deviations (SDs) were conducted for classifying the total actions between DMUs and plays. Second, the Kolmogorov–Smirnov test was performed. The homogeneity of variance was tested by the Levene test. According to the data obtained, an inferential analysis was carried out through parametric tests. To compare the average of the dependent variables (offensive performance) in each matched situational variable, we used the chi-square test. One-way analysis of variance was used to compare the averages of the dependent variables between duration and number of plays. Then, we conducted the *post hoc* Bonferroni test. Finally, a correlational analysis (Pearson’s rho) was used to estimate the relationships between number of players and age group.

The effect size (*d*) was calculated with the aim of interpreting the difference in the variables measured between the three age groups analyzed. The method used was Cohen’s *d*, as it is especially suitable for studies that adopt two-group designs, with the result being measured continuously by calculating the difference between the group means and, optionally, dividing it by the shared SD of both groups (Steenbergen-Hu and Olszewski-Kubilius, 2016). It was considered trivial (0–0.19), small (0.20–0.49), medium (0.50–0.79), and large (0.80 and greater) (Cohen, 1992), defined as follows:

$$d=\frac{{\bar{X}}_{1}-{\bar{X}}_{2}}{\sqrt{\left(s_{1}^{2}+s_{2}^{2}\right)/2}}$$

where ${\bar{X}}_{1}-{\bar{X}}_{2}$ is the means of the experimental and control groups at the end of the program, and *s* is the typical shared deviation of the two groups.

The confidence interval was 95%, and the *p*-value was established (*p* < 0.05). The analyses were performed in IBM SPSS Statistics software for Windows, v. 24.0.

## Results

The DM of a talented group of players was evaluated in the U-10, U-12, and U-14 age groups. The descriptive analysis revealed that of the total number of plays (324), the U-12 was the age group with the greatest number of plays (129), followed by U-10 (105), and U-14 (90). On the other hand, the results showed that U-12 took the longest time in each play (10.58 s), followed by U-14 (10.27 s) and finally U-10 (8.29 s).

On the other hand, the quantitative analysis revealed a total of 763 DMUs. A total of 323 DMUs were performed in U-12, followed by 236 in U-14 and 204 in U-10. Thus, it is possible to observe that in U-14, greater DMUs per play were produced (2.62 DMUs/play), followed by U-12 (2.50 DMUs/play) and U-10 (1.94 DMUs/play). We observed that U-14 is the age group where fewer plays are made, but with a greater number of DMUs per play.

Then, the chi-square statistic was applied between the number of plays and the number of DMUs. The results showed no significant differences between them (*p* = 0.883). With the objective of comparing the age groups in relation to the duration of the plays and number of DMUs, the one-factor ANOVA statistic was executed (see Table 3).

**TABLE 3:** One-factor ANOVA between the variables duration of plays and number of DMUs.

	**Sum of squares**	**DF**	***F***	***P***
Duration of plays	338.326	2	5.356	0.005
Number of DMUs	27.121	2	6.812	0.001

*F, value of the test statistic. DMUs, decision-making units. DF, degrees of freedom.*

In the *post hoc* Bonferroni test (see Table 4), it was noted that the differences between U-10 and U-12, and U-10 and U-14 were significant statistically in play duration (*p* = 0.006 and *p* = 0.044, respectively) and number of DMUs (*p* = 0.008 and *p* = 0.003, respectively). However, the difference between U-12 and U-14 did not show significance.

**TABLE 4:** Bonferroni test between the variable duration of plays and number of DMUs.

**Duration of plays × Number of DMUs**	**Difference of means**	**Standard error**	***p***	***d***
U-10–U-12	−2.296*	0.739	0.006	−0.421
U-10–U-14	1.981*	0.807	0.044	−0.355
U-10–U-12	−0.561*	0.185	0.008	−0.450
U-10–U-14	−0.679*	0.203	0.003	−0.458

*d, effect size. DMUs, decision-making units.*

The magnitude in the change is shown in Table 4 (*d*). Thus, the effect sizes showed a negative direction in the change with a small magnitude in all the cases.

For in-depth statistical analysis, a correlational analysis was carried out. The results showed the relation between the duration of the plays and the number of DMUs performed in each age group (*p* = 0.000), with a high association in U-10 (*r* = 0.687, large correlation) and U-12 (*r* = 0.702, very large correlation) and a moderate association in U-14 (*r* = 0.597, large correlation). In addition, a partial correlation analysis (see Table 5) was made by isolating the variable number of players. The results showed how the intensity of association in U-12 is the lowest; meanwhile, U-10 and U-14 have increased in comparison with the previous correlation.

**TABLE 5:** Partial correlation (Pearson’s rho) between duration of plays and number of DMUs isolating the variable number of players.

**Duration of plays × Number of DMUs**	***P***	***r***
U-10	0.000	0.706
U-12	0.000	0.601
U-14	0.000	0.754

*r, correlation coefficient. DMUs, decision-making units.*

Finally, regarding the analyzed effectiveness in both tactical principles and technical–tactical skills by the players, Table 6 summarizes the results obtained.

**TABLE 6:** Rate of success (%) of DMUs in each age group.

	**U-10**	**U-12**	**U-14**
**Tactical principles**			
1A (keeping)	82.45	87.38	90.24
2A (progressing)	89.54	96.11	96
3A (scoring)	100	100	100
**Technical–tactical skills**			
Passing	81.25	87.93	93.75
Dribbling	40	70.58	83.33
Shooting	–	85.74	100
Getting free	93.13	94.94	96.29
Variable offensive	90.47	90.16	97.46

*DMUs, decision-making units.*

Even though that in U-12 there are a greater number of plays (129) of longer duration (10.58 s), and also there are a greater number of DMUs (323), the effectiveness is higher in U-14 in all the tactical principles and technical–tactical skills, except for the 2A principle (progressing), which showed greater effectiveness in the U-12 age group. Thus, it is possible to observe how the effectiveness increased with age.

## Discussion

This study aimed to compare the DM of a group of talented players from U-10 to U-14. The NSIFT has been utilized for TI and the GPET for the analysis of the actions performed. On the one hand, it is important to note that there are no previous studies that had considered a TI process to compose the talent pool. For example, Sánchez-Mora et al. (2011) divided the sample into teams on the basis of the level of knowledge, but they utilized subjective measures for conducting the process. Hastie et al. (2011) used as participation criterion in the final sample having attended three testing conditions and six practice sessions. In the study of Araújo et al. (2016), the participants had no previous experience. On the other hand, the size of talented sample used here (*N* = 18) is bigger than that of previous studies. For example, in a recent study conducted by Práxedes et al. (2018), they selected 10 players with a high level of expertise as sample of their study.

The results found here confirm the importance of the CLA theory when designing task for each age group (Machado et al., 2019). Thus, previous studies have concluded the importance of the context (Travassos et al., 2017), for example, the use of different marks in the pitch (Coutinho et al., 2019) or number of players (Vilar et al., 2012). In this study, we compare the DMU in IG in each age group, depending on the number of players and size of the pitch. In the light of the CLA theory, the results found are discussed hereunder.

In relation to the descriptive analysis, the duration of the plays increased from U-10 to U-12 but decreased in U-14. However, the DMUs increased in this age group. It was observed that despite the decrease in the number of plays, there are greater DMUs in U-14. This fact can be explained by the greater skill acquired in older stages, owing to the expertise accumulated (Collins and MacNamara, 2012; Sarmento et al., 2018). This fact comes to conclude that game speed in IG increases in older age group. This result has been found, not only in the PE setting (Blomqvist et al., 2005) but also in soccer context (Murtagh et al., 2017).

In regard with the effectiveness of DM, the U-14 is the age group in which there is greater effectiveness, except for the principle of progressing (2A), which showed better scores in U-12. According to this last result, González-Víllora et al. (2015a) found that, in the first formation stages, the teaching of tactics focuses its attention on the progression, although it is in the U-12 and U-14 age groups where maintaining possession (1A) gains more weight, giving more importance to make more number of passes in this age group (Serra-Olivares et al., 2016).

Regarding the inferential analysis, the difference between duration of the play and number of DMUs was statistically significant between the U-10 and U-14 age groups. However, the U-12 age group did not score such differences. That leads to think about the need to review the progression in this age groups, with the purpose of adjusting the decisional demands to each age group (Castellano et al., 2013). In addition, in the U-12 age group, the relationship between plays and DMU is lower than in U-10 and U-14. Thus, we can observe that the progression of DMUs per play is not followed in the U-12 age group. In relation with this result, González-Víllora et al. (2015a) concluded 4 years ago that the U-12 did not follow the progression in the decisional demands among U-10 and U-14. In addition, Castellano et al. (2016) focused their research on U-12 players, concluding that some large-side games could be avoided because the physical and physiological demands are not adjusted to the maturation of these players. Finally, Martone et al. (2017) evaluated the effects of six different area per player on exercise intensity and technical actions in U-12 and U-14 soccer players during small-sided games. They concluded that U-14 players adapted better to area per player changes. It is emphasized, therefore, that the organizations responsible for managing the competitions must be aware that in U-12, the progression in the decisional demands does not seem to be suitable for children with this age, so it would be necessary to review the organizational aspects in this age (number of players or size of the field) to create a progression more adjusted to their needs. Thus, future studies must be focused on researching if the number of players or the size of the pitch in U-12 might be changed, in order to follow the relationship between DMUs per play from U-10 to U-14.

On the other hand, as a research prospective, the results found in this work can be the starting points of different investigation lines related to the adequate progression of decisional demands in the formative stages of the young player in IG. For example, it would be necessary to emphasize that future studies should focus their objectives on keeping this work with the U-16, U-18, and U-23 age groups, with the purpose of knowing the effectiveness, duration, and number of DMUs in older age groups.

Regarding teaching and learning process, policy makers must put all the new trends into practice. They must include in the official curriculum a framework about how to deal with talented children in both educational and soccer contexts. These results, along with the CLA theory (Renshaw et al., 2012) and the different models of sport initiation (Araújo et al., 2016) that fits better in PE to deal with talented children, such as the multifunctional pathways model (Culpan, 2005), the alternative model (Kirk and Gorely, 2000) or the sport education model (Siedentop, 1994) will make a better teaching and learning process for talented children in sports.

Finally, this work presents some limitations. First, only one team took part in the study. Second, no female participants were included. Third, it would be recommendable to increase the sample size. These limitations must be taken into account in future studies.

## Conclusion

Once the study has been conducted, we concluded the following points. First, there is progressive acquisition of expertise as the age group increased, as well as a better percentage of success in DM in older age groups. Second, the game speed increases with age, making more DMUs and lower number of plays. Third, the effectiveness has to be more than 80% for children to be considered as talented. Finally, it is concluded that there is a necessity of reviewing the organizational aspects in the U-12 age group, related mainly to the size of the pitch and the number of players, because it does not follow the same progression in regard to decisional demands.

## Data Availability Statement

The datasets generated for this study are available on request to the corresponding author.

## Ethics Statement

Prior to data collection, we informed the participants and their parents about the research aims, procedures, risks, and benefits of the study, and we obtained the required consent from the parents of the players. Participation was voluntary with the parents’ consent, and the anonymity of the participants and data confidentiality was guaranteed during the whole process. The study conformed to the deontological guidelines defined by the Declaration of Helsinki (Hong Kong Revision, 1989) and followed the recommendations of the Good Clinical Practice in the European Community (1 July 1991, document 111/3976/88) and the Spanish legislation on human clinical research (Royal Decree 561/1993 on clinical trials).

## Author Contributions

JP-V contributed to the design, introduction, methodology, results, discussion, and conclusion. AP-A contributed to the introduction, methodology, results, and conclusion. OC-J contributed to the design and methodology. FC, PN, TR, and BK drafted, revised, and approved the final version.

## Conflict of Interest

AP-A was employed by Albacete Balompiè S.A.D.

The remaining authors declare that the research was conducted in the absence of any commercial or financial relationships that could be construed as a potential conflict of interest.
